# Anti-aging protein α-Klotho is potential for reducing comorbidity risk of cardiometabolic diseases in vulnerable populations and enhancing long-term prognosis

**DOI:** 10.1038/s41598-025-01580-4

**Published:** 2025-05-14

**Authors:** Kai Wang, Jianing Liu

**Affiliations:** 1 Medical School, Southeast University, Nanjing, China; 2 Medical Faculty, Ulm University, Ulm, Germany

**Keywords:** α-Klotho, Cardiometabolic diseases, Prevalence, All-cause mortality, Population-specificity, Risk factors, Cardiovascular biology, Epidemiology, Population screening

## Abstract

This study investigated the impact of anti-aging protein α-Klotho on cardiometabolic diseases (CMDs) among middle-aged and elderly population. A total of 11,198 participants aged 40–79 years were included in the National Health and Nutrition Examination Survey (NHANES) spanning 2007–2016. Serum α-Klotho levels were quantified via enzyme-linked immunosorbent assays. CMDs comprised cardiovascular disease (CVD), and four metabolic disorders: type 2 diabetes (T2DM), obesity, chronic kidney disease (CKD), and non-alcoholic fatty liver disease (NAFLD). Weighted logistic regression analysis, subgroup analysis, mediation analysis, restricted cubic splines (RCS), and Cox proportional hazards regression analysis were used. α-Klotho exhibited negative associations with each single CMD except T2DM, and RCS showed U-shape and L-shape dose-response relationships of α-Klotho with risk of T2DM and CKD, respectively. Ordered logistic regression analysis revealed that higher levels of Klotho markedly reduced the cumulative number of metabolic comorbidities complicating CVD (OR 0.56 (0.35, 0.91)). Simple mediation analysis showed CKD may explain up to 20.42% of the association between Klotho and CVD. Notably, α-Klotho’s association with cardiometabolic comorbidities was particularly evident among individuals who were widowed/divorced/separated, non-Hispanic Black, lower-income, or less educated, with hypertension, current smokers, lower leisure and commuting physical activity, but higher work-related physical activity. Regarding long-term effects, higher α-Klotho levels were associated with lower all-cause mortality among participants with CMDs, but not among those without CMDs. Higher α-Klotho levels were associated with lower CMD prevalence, particularly in high-risk cardiovascular populations with lower socioeconomic status and unfavorable lifestyles and reduced all-cause mortality risk among CMD patients.

**Supplementary Information:**

The online version contains supplementary material available at 10.1038/s41598-025-01580-4.

## Introduction

Although early intervention and advancements in medical technology have contributed to a slowdown in the prevalence and mortality rates of cardiovascular disease (CVD)^1^, it remains one of the leading causes of mortality among adults, particularly those in middle age and the elderly^2,3^. It’s essential to recognize that solely focusing on clinical monitoring and intervention for cardiovascular disease (CVD) as an isolated condition is impractical. CVD rarely occurs in isolation; instead, it frequently coexists with specific metabolic disorders. These disorders are closely intertwined and collectively termed cardiometabolic diseases (CMD). Defining the scope of cardiometabolic diseases has lacked uniformity, but in 2023, the European Society of Cardiology identified type 2 diabetes (T2DM), obesity, chronic kidney disease (CKD), and non-alcoholic fatty liver disease (NAFLD), together with CVD, as the most prevalent CMDs^4^.

With the global trend of population aging, the increasing prevalence of cardiometabolic comorbidities is unsurprising, given the overlapping etiology and bidirectional interactions among most cardiometabolic diseases^5,6^. The concurrent presence of cardiovascular disease (CVD) and metabolic abnormalities frequently correlates with significantly worse survival outcomes. Globally, approximately 50% of deaths among individuals with type 2 diabetes mellitus (T2DM) are attributed to CVD^7^. High body mass index (BMI) accounted for 5.0 million deaths and 160 million disability-adjusted life-years (DALYs) of which more than half were due to CVD^8^. Moreover, BMI may not adequately capture the diverse obesity phenotypes and their respective risk profiles^9^; therefore, considering factors like waist circumference may offer better markers of adiposity distribution, which is also crucial for identifying cardiometabolic status^10^. Besides, patients with CKD and CVD have a remarkably higher mortality rate (58–71%), in contrast to those with CVD alone and normal renal function (22-27.5%)^11^. Several cohort studies also indicated a heightened risk of cardiovascular mortality among patients with NAFLD^12–14^. However, clinical practice and research tend to primarily focus on diseases of the heart itself, often overlooking the frequent coexistence of metabolic comorbidities with cardiovascular disease. This oversight results in limited research addressing the systemic concept of cardiometabolic diseases as a focal point.

On the other hand, due to the close association between numerous prevalent chronic progressive diseases and age, there is increasing attention on exploring the impact of aging-related factors. In 1997, Kuro-o, M. and colleagues reported a novel gene knockout mouse model that exhibited shortened lifespan, infertility, atherosclerosis, mottled skin, osteoporosis, and emphysema. This gene was identified as an aging suppressor and was named Klotho^15^. The primary isoform, α-Klotho, emerged as the pioneer member of the Klotho family, categorized into membrane-bound, secreted, and circulating soluble forms^16^. Soluble Klotho exists in blood, urine, and cerebrospinal fluid and plays various roles, including the regulation of ion channels/transporters and growth factor signaling pathways, serving as a circulating hormone^17,18^. While Klotho has been investigated concerning CVD in several studies^19^, it’s important to highlight that, to date, there hasn’t been a comprehensive exploration yielding conclusive evidence regarding the impact of Klotho on cardiometabolic comorbidities. Furthermore, it remains unknown whether the influence of Klotho varies among specific populations. Given the pivotal role of cardiometabolic diseases in influencing survival outcomes, there is a pressing need for clinical reports to elucidate potential long-term prognosis differences between patients with CMDs and non-diseased individuals.

In light of the aforementioned gaps, this study aims to provide a comprehensive and nuanced investigation for unraveling the specific associations between Klotho and the prevalence of CMDs in middle-aged and elderly individuals, while also exploring potential population-specific effects, and shedding light on the yet-unexplored interplay between Klotho and the long-term prognosis of individuals affected by CMDs.

## Methods

### Data source and study population

This study employed data from the National Health and Nutrition Examination Survey (NHANES) conducted between 2007 and 2016. NHANES is a thorough national survey managed by the National Centre for Health Statistics (NCHS) in the United States. It follows a multistage, stratified, subgroup probability sampling design in two-year cycles. The participant selection process is illustrated in Supplementary Fig. 1. Regarding inclusion criteria, only adult subjects aged 40–79 years with complete α-Klotho information were included. Subjects without complete information of CMDs (including CVD, T2DM, general obesity, central obesity, CKD, and NAFLD) and those with missing covariates were excluded. Ultimately, the study included 11,198 eligible participants with complete data.

### Exposure variable: α-Klotho measurement

Serum specimens collected in NHANES were preserved at -80 °C until undergoing Klotho analysis. Klotho levels were measured using enzyme-linked immunosorbent assay (ELISA) kits manufactured by IBL International, Japan. Each sample underwent analysis twice, with the results automatically transferred to the laboratory’s Oracle management system for assessment by trained supervisors. If the duplicate results deviated by more than 10%, the analysis was considered invalid, necessitating a repeat sample analysis.

### Outcome variables: cmds

Cardiometabolic abnormalities are defined based on established criteria for cardiovascular disease (CVD), Type 2 diabetes mellitus (T2DM), obesity (both general and central), chronic kidney disease (CKD), and non-alcoholic fatty liver disease (NAFLD). CVD was defined in our study as any reported diagnosis of CVD (heart failure/coronary heart disease/angina pectoris/heart attack/stroke)^20^. T2D was defined as fasting plasma glucose (FPG) ≥ 7.0 mmol/L (126 mg/dL), oral glucose tolerance test two hour plasma glucose ≥ 11.1 mmol/L (200 mg/dL), glycosylated hemoglobin (HbA1c) ≥ 6.5%, self-reported of T2D, or currently receiving hypoglycemic therapy^21,22^. The classification of general obesity followed World Health Organization guidelines, stratifying individuals into normal weight (18.5 kg/m² ≤ BMI < 25 kg/m²), overweight (25 kg/m² ≤ BMI < 30 kg/m²), and obese (BMI ≥ 30 kg/m²)^23^. Central obesity was identified by WC exceeding 102 cm for males or 88 cm for females^24^. According to the previous literature, the Chronic Kidney Disease Epidemiology Collaboration (CKD-EPI) equation was used to estimate the glomerular filtration rate, named eGFR^25^. CKD is defined by urinary albumin-to-creatinine ratio (≥ 30 mg/g or 3 mg/mmol) and eGFR(<60 mL/min/1.73 m^2^)^26^. The gold standard for diagnosing NAFLD is liver biopsy, but it is not practical in general population studies due to the invasiveness and high costs of the procedure. hepatic steatosis index (HSI) is a common non-invasive screening tool to predict. NAFLD presence is calculated based on the following specific formula = 8 × alanine aminotransferase (ALT, IU/L)/aspartate aminotransferase (AST, IU/L) + body mass index (BMI, kg/m^2^) + 2 (if female) + 2 (if type 2 diabetes). Furthermore, participants were defined as having NAFLD when HSI > 36 according to a previous publication^27^.

### Assessment of mortality

The vital status of participants was determined by linking their records to the National Death Index (NDI), allowing for the identification of deceased individuals. Data on all-cause mortality were collected until December 31, 2019, utilizing the 2019 Linked Mortality File (LMF), which includes the latest linkages between specific NCHS surveys and the NDI.

### Covariates

The chosen covariates, previously linked to Klotho and cardiometabolic diseases in prior studies, comprised: (1)Demographic data: age, sex, race, marriage status, education level, and family poverty income ratio (PIR); (2) Lifestyle factors: smoking status, alcohol consumption, sedentary behavior, and vigorous/moderate physical activity (V/MPA) of diverse life domains, containing recreational activities (leisure-time), work-related (occupational), and commuting, respectively; (3)Physical examination: systolic blood pressure (SBP, mmHg), diastolic blood pressure(DBP, mmHg), body mass index(BMI, kg/m^2^), and waist circumference(WC, cm); (4)Medical history: hypertension, and cancer history.

V/MPA was categorized based on engagement in activities causing significant sweating or a substantial increase in respiration or heart rate during a typical week^28^. Sedentary behavior was considered as remaining in a seated position for > 7.5 h per day^29^. Smoking status was delineated as follows: never smoker (smoked less than 100 cigarettes in life); former smoker (smoked at least 100 cigarettes in life and not at all now); current smoker (smoked at least 100 cigarettes in life and smoked some days or every day now)^30^. The alcohol consumption of participants was classified into four types: Non-drinker, 1–5 drinks/month, 5–10 drinks/month, and 10 + drinks/month^31^. Hypertension was defined per the American Heart Association Blood Pressure Guidelines, incorporating systolic blood pressure (SBP) ≥ 140 mmHg and/or diastolic blood pressure (DBP) ≥ 90 mmHg, self-reported hypertension, or the use of antihypertensive medication^32^.

## Results

### Characteristics of study participants

In this study, a total of 11,198 participants were included, and the weighted prevalence of cardiovascular disease (CVD) was 12.87%. Table 1 provides a comprehensive overview of the basic characteristics of the study participants, emphasizing notable distinctions between the CVD and non-CVD groups concerning demographics, lifestyle factors, medical history, and the prevalence of cardiometabolic diseases. Examining the demographic characteristics, the CVD group was predominantly male (58.53%), older in age, with higher levels of poverty and lower educational attainment compared to the non-CVD group. Regarding lifestyle habits, a higher proportion of smokers and lower levels of vigorous/moderate physical activity (VMPA) were evident in the CVD group. Furthermore, it was noteworthy that the serum level of soluble α-Klotho was significantly lower in the CVD group compared to the non-CVD group.

**Table 1:** Basic characteristics of the study participants

Characteristic	Overall, N^1^ = 11198 (100%)^2^	Non-CVD, N = 9757 (87.13%)^2^	CVD, N = 1441 (12.87%)^2^	P Value^3^
Age (years)	55.0 (47.0, 64.0)	54.0 (47.0, 63.0)	65.0 (57.0, 71.0)	< 0.001
Family PIR	3.51 (1.79, 5.00)	3.67 (1.91, 5.00)	2.23 (1.20, 4.31)	< 0.001
Sex				< 0.001
Female	5,709.00 (51.79%)	5,113.00 (52.97%)	596.00 (41.47%)	
Male	5,489.00 (48.21%)	4,644.00 (47.03%)	845.00 (58.53%)	
Race				0.006
Non-Hispanic White	5,073.00 (74.85%)	4,352.00 (74.97%)	721.00 (73.79%)	
Non-Hispanic Black	2,204.00 (8.78%)	1,875.00 (8.51%)	329.00 (11.08%)	
Mexican American	1,722.00 (6.25%)	1,561.00 (6.43%)	161.00 (4.64%)	
Other Hispanic	1,215.00 (4.28%)	1,073.00 (4.32%)	142.00 (3.88%)	
Other/multiracial	984.00 (5.85%)	896.00 (5.76%)	88.00 (6.60%)	
Marriage status				0.007
Married/Living with Partner	7,271.00 (70.61%)	6,413.00 (71.08%)	858.00 (66.53%)	
Widowed/Divorced/Separated	3,018.00 (22.29%)	2,543.00 (21.77%)	475.00 (26.75%)	
Never married	909.00 (7.10%)	801.00 (7.15%)	108.00 (6.72%)	
Education Level				< 0.001
Above high school	5,755.00 (62.44%)	5,159.00 (63.82%)	596.00 (50.44%)	
High school	2,497.00 (22.12%)	2,140.00 (21.68%)	357.00 (25.98%)	
Below high school	2,946.00 (15.44%)	2,458.00 (14.51%)	488.00 (23.58%)	
Hypertension	6,020.00 (48.13%)	4,880.00 (44.95%)	1,140.00 (75.74%)	< 0.001
Cancer	1,353.00 (13.74%)	1,081.00 (12.90%)	272.00 (21.08%)	< 0.001
Smoking status				< 0.001
Current smoker	2,210.00 (18.29%)	1,828.00 (17.39%)	382.00 (26.05%)	
Former smoker	3,316.00 (30.35%)	2,764.00 (29.14%)	552.00 (40.82%)	
Never smoker	5,672.00 (51.37%)	5,165.00 (53.46%)	507.00 (33.13%)	
Alcohol consumption				< 0.001
Non-drinker	3,196.00 (21.83%)	2,785.00 (21.59%)	411.00 (23.90%)	
1–5 drinks/month	5,465.00 (48.99%)	4,676.00 (48.16%)	789.00 (56.16%)	
5–10 drinks/month	752.00 (8.31%)	690.00 (8.81%)	62.00 (4.01%)	
10 + drinks/month	1,785.00 (20.87%)	1,606.00 (21.43%)	179.00 (15.93%)	
Recreational VMPA	4,990.00 (51.43%)	4,510.00 (53.00%)	480.00 (37.78%)	< 0.001
Work VMPA	4,403.00 (43.52%)	3,883.00 (43.76%)	520.00 (41.38%)	0.2
Commuting VMPA	2,531.00 (20.85%)	2,263.00 (21.49%)	268.00 (15.31%)	< 0.001
Sedentary Behavior	3,649.00 (37.69%)	3,151.00 (37.44%)	498.00 (39.89%)	0.11
Type 2 Diabetes	2,819.00 (18.96%)	2,164.00 (16.39%)	655.00 (41.27%)	< 0.001
General Obesity	4,677.00 (40.25%)	3,932.00 (38.74%)	745.00 (53.36%)	< 0.001
Central Obesity	7,214.00 (64.68%)	6,164.00 (63.57%)	1,050.00 (74.32%)	< 0.001
CKD	2,221.00 (15.97%)	1,655.00 (13.82%)	566.00 (34.61%)	< 0.001
NAFLD	6,915.00 (59.67%)	5,930.00 (58.63%)	985.00 (68.73%)	< 0.001
Serum α-Klotho (pg/ml)	798.00 (657.00, 977.10)	801.04 (660.43, 980.20)	773.88 (627.92, 947.01)	< 0.001

^1^N not Missing (unweighted)

^2^ medians (IQR, 25th percentage, 75th percentage) for continuous; n (%) for categorical

^3^ Wilcoxon rank-sum test for complex survey samples; chi-squared test with Rao & Scott’s second-order correction

### Linear and non-linear relationships between α-Klotho and prevalence of each single cardiometabolic disease

To elucidate the relationship between α-Klotho and individual diseases of cardiometabolic diseases, binary logistic regression was employed in this study. As shown in Table 2, after full adjustment (Model 3), significant associations were observed between α-Klotho and CVD, general obesity, central obesity, CKD, and NAFLD. Specifically, higher levels of α-Klotho were associated with a reduced risk of the above cardiometabolic diseases, indicating a protective effect (For CVD: OR 0.60 (0.38, 0.97); For General Obesity: OR 0.55 (0.39, 0.78); For Central Obesity: OR 0.44 (0.29, 0.66); For CKD: OR 0.34 (0.19, 0.60); For NAFLD: OR 0.31 (0.21, 0.46)). Although there was no significant association between α-Klotho and T2DM, using binary logistic regression (OR 1.03 (0.64, 1.66), *P* = 0.9), a remarkable non-linear relationship was found shown in Supplementary Fig. 2B. In addition, the results of trend tests for quartiles of log10-transformed Klotho demonstrate that, when considering general obesity, central obesity, CKD, and NAFLD as outcome variables, the trend P-values are all less than 0.05. This indicates that a higher quartile of serum Klotho is associated with a stronger protective effect against cardiometabolic diseases compared to the lowest quartile of serum Klotho. This study further investigated the relationship between α-Klotho levels and traditional cardiovascular risk markers, including glycated hemoglobin, C-reactive protein, LDL, and HDL cholesterol. The findings are presented in Supplementary Table 1.

**Table 2:** The associations between α-Klotho and cardiometabolic diseases using binary logistic regression

Outcomes		Model 1		Model 2		Model 3	
		OR (95%CI)	P value	OR (95%CI)	P value	OR (95%CI)	P value
CVD	log_10_(α-Klotho)	0.34 (0.21, 0.57)	< 0.001	0.58 (0.35, 0.96)	0.035	0.60 (0.38, 0.97)	0.036
	log_10_(α-Klotho) (Quartile)						
	Q1	Reference		Reference		Reference	
	Q2	0.76 (0.61, 0.95)	0.018	0.83 (0.65, 1.06)	0.13	0.84 (0.66, 1.08)	0.2
	Q3	0.78 (0.64, 0.94)	0.011	0.90 (0.73, 1.11)	0.3	0.92 (0.74, 1.15)	0.5
	Q4	0.69 (0.56, 0.85)	< 0.001	0.83 (0.66, 1.03)	0.092	0.82 (0.66, 1.02)	0.067
	P for trend	0.001		0.15		0.12	
T2DM	log_10_(α-Klotho)	0.97(0.63, 1.49)	0.9	1.30 (0.85, 1.99)	0.2	1.03 (0.64, 1.66)	0.9
	log_10_(α-Klotho) (Quartile)						
	Q1	Reference		Reference		Reference	
	Q2	0.90 (0.76, 1.08)	0.3	0.97 (0.80, 1.17)	0.7	0.96 (0.79, 1.16)	0.7
	Q3	0.93 (0.80, 1.08)	0.3	1.03 (0.88, 1.21)	0.7	1.00 (0.84, 1.17)	> 0.9
	Q4	1.06 (0.90, 1.24)	0.5	1.18 (1.00, 1.39)	0.53	1.07(0.90, 1.28)	0.4
	P for trend	0.5		0.046		0.4	
General Obesity	log_10_(α-Klotho)	0.78 (0.55, 1.08)	0.14	0.75 (0.54, 1.04)	0.087	0.55 (0.39, 0.78)	0.001
	log_10_(α-Klotho) (Quartile)						
	Q1	Reference		Reference		Reference	
	Q2	0.98 (0.84, 1.13)	0.7	0.99 (0.85, 1.15)	0.9	0.98 (0.83, 1.15)	0.8
	Q3	0.94 (0.81, 1.09)	0.4	0.96 (0.83, 1.11)	0.6	0.91 (0.78, 1.06)	0.2
	Q4	0.94 (0.82, 1.07)	0.3	0.93 (0.81, 1.06)	0.3	0.82 (0.72, 0.94)	0.004
	P for trend	0.3		0.2		0.003	
Central Obesity	log_10_(α-Klotho)	0.58 (0.39, 0.87)	0.009	0.57 (0.37, 0.88)	0.011	0.44 (0.29, 0.66)	< 0.001
	log_10_(α-Klotho) (Quartile)						
	Q1	Reference		Reference		Reference	
	Q2	0.89 (0.77, 1.03)	0.11	0.93 (0.80, 1.08)	0.3	0.91 (0.77, 1.09)	0.3
	Q3	0.86 (0.75, 0.98)	0.86	0.90 (0.79, 1.02)	0.10	0.86 (0.75, 0.98)	0.027
	Q4	0.83 (0.71, 0.96)	0.016	0.83 (0.71, 0.97)	0.020	0.75 (0.64, 0.87)	< 0.001
	P for trend	0.011		0.016		< 0.001	
CKD	log_10_(α-Klotho)	0.25 (0.14, 0.44)	< 0.001	0.34 (0.19, 0.60)	< 0.001	0.34 (0.19, 0.60)	< 0.001
	log_10_(α-Klotho) (Quartile)						
	Q1	Reference		Reference		Reference	
	Q2	0.75 (0.63, 0.90)	0.002	0.80 (0.66, 0.98)	0.028	0.81 (0.67, 0.98)	0.030
	Q3	0.64 (0.51, 0.79)	< 0.001	0.70 (0.55, 0.88)	0.003	0.70 (0.55, 0.89)	0.004
	Q4	0.62 (0.51, 0.76)	< 0.001	0.69 (0.56, 0.86)	0.001	0.68 (0.54, 0.85)	< 0.001
	P for trend	< 0.001		< 0.001		< 0.001	
NAFLD	log_10_(α-Klotho)	0.48 (0.33, 0.71)	< 0.001	0.46 (0.31, 0.68)	< 0.001	0.31 (0.21, 0.46)	< 0.001
	log_10_(α-Klotho) (Quartile)						
	Q1	Reference		Reference		Reference	
	Q2	0.88 (0.77, 1.00)	0.058	0.89 (0.77, 1.02)	0.085	0.86 (0.74, 1.00)	0.057
	Q3	0.87 (0.75, 1.01)	0.060	0.87 (0.75, 1.01)	0.069	0.81 (0.70, 0.95)	0.009
	Q4	0.80 (0.69, 0.93)	0.004	0.79 (0.68, 0.91)	0.002	0.68 (0.59, 0.79)	< 0.001
	P for trend	0.006		0.004		< 0.001	

Model 1: Univariate model

Model 2: Adjusted for age, sex, race/ethnicity, marital status, education level, and poverty income ratio (PIR)

Model 3: Additional adjustments included hypertension history, cancer history, alcohol consumption, smoking status, recreational VMPA, work VMPA, commuting VMPA, and sedentary behavior.

Furthermore, we utilized Restricted Cubic Splines (RCS) to explore the potential nonlinear dose-response relationships between α-Klotho and cardiometabolic diseases (Supplementary Figure Fig. 2A and F). While no significant nonlinear relationships were observed between Klotho and CVD, general obesity, central obesity, and NAFLD, notable nonlinear associations were identified between Klotho and T2DM and CKD (P for non-linear < 0.05). These relationships exhibited a U-shaped and L-shaped pattern, respectively. For T2DM and CKD outcomes, when log-transformed Klotho levels were below 2.67, the risk of T2DM and CKD increased. However, as log-transformed Klotho exceeded 2.67, the risk of T2DM and CKD decreased, indicating a protective role of Klotho. Notably, for CKD, this significant protective effect disappeared when log-transformed Klotho exceeded 3.30, revealing an overall L-shaped relationship between the independent variable and the outcome. In contrast, the protective effect of log-transformed Klotho for T2DM was only evident in the range of 2.67 to 2.90. Beyond this range, with increasing levels of Klotho, this protective effect became less apparent. Even when log-transformed Klotho exceeded 3.16, Klotho became a significant risk factor for T2DM, demonstrating a U-shaped relationship.

### Associations of α-Klotho with concurrent risk of cardiometabolic diseases

As to cardiometabolic diseases in the concurrent status, the results from ordered logistic regression (Table 3) suggest that serum α-Klotho exhibits a significant reduction effect against the risk of CVD complicated with any cardiometabolic comorbidities (all *P* < 0.05). Moreover, RCS plots (Supplementary Fig. 2G to Fig. 2J) demonstrate that there was no significant non-linear relationship between α-Klotho and the prevalence of cardiometabolic comorbidities (all P for nonlinear > 0.05). To address the impact of Klotho on reducing the number of metabolic comorbidities complicating CVD, we used “CVD complicated with increased metabolic comorbidities” as an ordered multicategory outcome variable, with a higher rank indicating more comorbidities. In the fully adjusted model, the odds ratio (OR) was 0.56, indicating that with each unit increase in log10(α-Klotho), there was a decreasing trend in the number of metabolic comorbidities accompanying CVD, with a magnitude of 0.56 times.

**Table 3:** The association between α-Klotho (log10-transformed) and CVD complicated with metabolic comorbidities using ordered logistic regression

Outcomes	Model 1*		Model 2*		Model 3*	
	OR (95%CI)	P value	OR (95%CI)	P value	OR (95%CI)	P value
CVD complicated with increased metabolic comorbidities ^#1^	0.34 (0.21, 0.56)	< 0.001	0.57 (0.34, 0.93)	0.026	0.56 (0.35, 0.91)	0.018
CVD complicated with T2DM^#2^	0.34 (0.21, 0.57)	< 0.001	0.60 (0.36, 0.99)	0.044	0.61 (0.38, 0.99)	0.044
CVD complicated with Obesity^#3^	0.35 (0.21, 0.57)	< 0.001	0.58 (0.35, 0.96)	0.035	0.60 (0.37, 0.97)	0.037
CVD complicated with CKD^#4^	0.33 (0.20, 0.54)	< 0.001	0.55 (0.34, 0.88)	0.014	0.55 (0.36, 0.85)	0.007
CVD complicated with NAFLD^#5^	0.34 (0.21, 0.56)	< 0.001	0.57 (0.35, 0.94)	0.028	0.59 (0.37, 0.94)	0.026

*Model 1: Univariate model

*Model 2: Adjusted for age, sex, race/ethnicity, marital status, education level, and poverty income ratio (PIR)

*Model 3: Additional adjustments included hypertension history, cancer history, alcohol consumption, smoking status, recreational VMPA, work VMPA, commuting VMPA, and sedentary behavior.

^#^The Outcome variables‘CVD complicated with increased metabolic comorbidities’, ‘CVD complicated with T2DM’, ‘CVD complicated with Obesity’, ‘CVD complicated with CKD’, and ‘CVD complicated with NAFLD’ are ordered multicategory variables.

^# 1^ The rank order of the five categorical items in the variable 'CVD complicated with increased metabolic comorbidities’: CVD with Four Metabolic Comorbidities > CVD with Three Metabolic Comorbidities > CVD with Two Metabolic Comorbidities > CVD with One Metabolic Comorbidity > CVD Without Any metabolic comorbidities > non-CVD.

^# 2^ The rank order of the three categorical items in the variable 'CVD complicated with T2DM’: CVD complicated with T2DM > CVD complicated without T2DM > non-CVD.

^# 3^ The rank order of the three categorical items in the variable 'CVD complicated with Obesity’: CVD complicated with Obesity (General or Central) > CVD complicated without Obesity > non-CVD.

^# 4^ The rank order of the three categorical items in the variable 'CVD complicated with CKD: CVD complicated with CKD > CVD complicated without CKD > non-CVD.

^# 5^ The rank order of the three categorical items in the variable 'CVD complicated with NAFLD’: CVD complicated with NAFLD > CVD complicated without NAFLD > non-CVD.

### Subgroup analysis: population-specific advantages in the association of α-Klotho and prevalence of cardiometabolic comorbidities

**Fig. 1:**
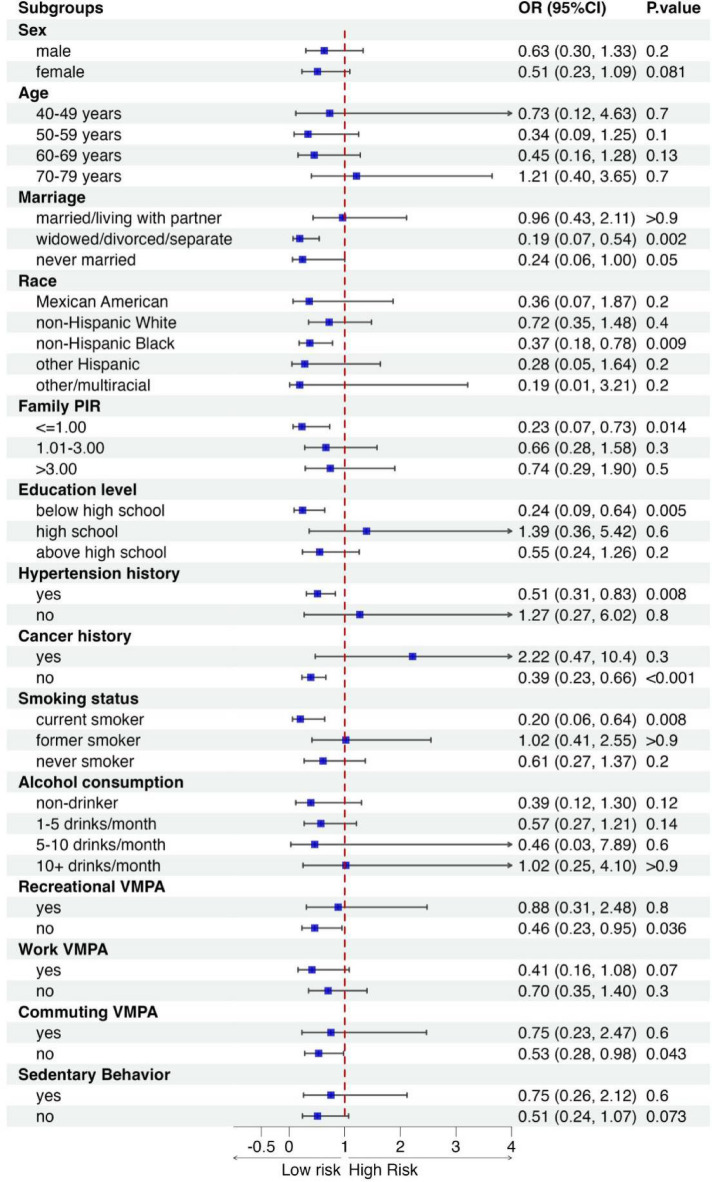
Forest plot for stratified analysis of the association between α-Klotho and cardiometabolic comorbidities ^#^. ^#^Adjusted for all factors in full adjustment model except the stratification variable.

To investigate whether the association between Klotho and cardiometabolic abnormalities varies in populations with different characteristics, we conducted a stratified analysis using demographic characteristics, lifestyle, and medical history as population segmentation factors. The outcome variable was defined as CVD complicated with metabolic abnormalities, indicating the presence of any one of the metabolic diseases, including T2DM, general obesity, central obesity, CKD, or NAFLD, in addition to CVD. The forest plot (Fig. 1) indicates that the association between higher Klotho levels and lower cardiometabolic comorbidity burden was more pronounced in specific subgroups with distinct characteristics. We could thus give a rough sociodemographic picture of this group of people: they might be widowed, divorced, or separated status, belong to the non-Hispanic Black ethnicity, have a family income below the poverty line (family PIR ≤ 1), possess a lower educational level (below high school), have a history of hypertension without a cancer history, are current smokers, and exhibit lower levels of moderate-to-vigorous physical activity during leisure and commuting but engage in higher levels during working hours.

### Mediation analysis: the effect of metabolic abnormalities on the association between α-Klotho and CVD

**Fig. 2:**
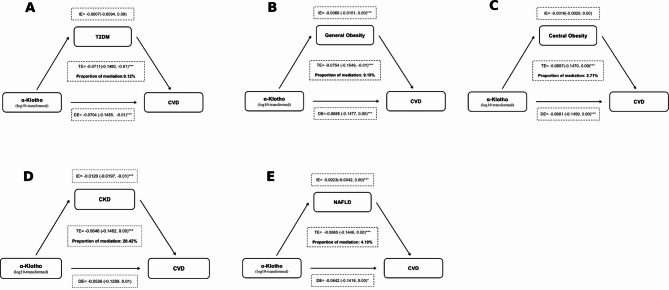
Mediation effect diagram of the effect of T2DM, obesity, CKD, or NAFLD on the association between α-Klotho and CVD^#^. CVD: cardiovascular disease; T2DM: Type 2 diabetes mellitus; CKD: chronic kidney disease; NAFLD: non-alcoholic fatty liver disease; DE: direct effect; IE: indirect effect; TE: total effect. (*: *P* < 0.05; **: *P* < 0.01; ***: *P* < 0.001). ^#^Adjusted for all factors in the full adjustment model.

It is known that CVD and metabolic abnormalities often occur concurrently, with complex interwoven relationships. They not only share certain pathogenic mechanisms to some extent but also mutually influence each other’s development. Given the observed negative association between α-Klotho and both CVD risk and cardiometabolic comorbidities, we conducted mediation analyses to explore whether metabolic conditions—T2DM, obesity, CKD, and NAFLD—serve as intermediaries in this relationship. In the simple mediation model (Fig. 2), significant indirect effects were observed for general obesity (9.19%), NAFLD (4.19%), and CKD (20.42%), indicating that CKD accounted for the largest proportion of Klotho’s protective association with CVD. Moreover, multiple mediation analysis (Supplement Fig. 3) provided deeper insights into the interconnections among metabolic disorders. The indirect pathways ‘α-Klotho → NAFLD → T2DM → CVD’ (*p* < 0.001) and ‘α-Klotho → NAFLD → T2DM → CKD → CVD’ (*p* < 0.001) demonstrated statistically significant mediation effects, suggesting that higher α-Klotho levels are inversely associated with this pathogenic cascade. Specifically, α-Klotho may help mitigate NAFLD-driven insulin resistance, thereby reducing the risk of T2DM, alleviating CKD burden, and ultimately lowering CVD risk. Additionally, the pathways ‘α-Klotho → obesity → T2DM → CVD’ (*p* = 0.064) and ‘α-Klotho → obesity → CKD → CVD’ (*p* = 0.061) showed borderline significance, implying a potential but less pronounced mediation effect.

### Survival analysis: the impact of α-Klotho on all-cause mortality risk in participants with and without cmds

To explore the long-term effects of the relationship between Klotho and cardiometabolic diseases, specifically, whether this association influences survival outcomes, we utilized data from the NHANES prospective cohort. The population was stratified based on the presence or absence of cardiometabolic diseases. Cox proportional hazards regression was employed with all-cause mortality as the outcome variable (Table 4). For individual diseases, in the fully adjusted model, higher Klotho levels were significantly associated with lower overall mortality risk only in individuals with CVD, general obesity, and CKD (HR (95% CI): CVD, 0.34 (0.13, 0.85); general obesity, 0.38 (0.17, 0.82); CKD, 0.42 (0.20, 0.90)). In contrast, there was no significant association between Klotho and all-cause mortality in non-afflicted populations with these diseases. Regarding combination status, there was a significant negative association between Klotho and the hazard ratio (HR) of all-cause mortality in populations with the comorbidities of CVD and T2DM, CVD and obesity, CVD and CKD with HRs (95%CI) of 0.23 (0.06, 0.93), 0.20 (0.06, 0.70) and 0.25 (0.05, 0.97), respectively. Conversely, in non-afflicted populations, the association between Klotho and mortality risk diminished, with HRs of 0.73 (0.40, 1.32), 0.79 (0.43, 1.45) and 0.75 (0.40, 1.40), respectively.

**Table 4:** The associations between α-Klotho(log10-transformed) and risk of all-cause mortality among participants with or without cardiometabolic diseases.

Outcomes		Model 1		Model 2		Model 3	
		HR (95%CI)	P value	HR (95%CI)	P value	HR (95%CI)	P value
CVD	Afflicted	0.32 (0.11, 0.93)	0.035	0.32 (0.13, 0.81)	0.017	0.34 (0.13, 0.85)	0.021
	Non-afflicted	0.45 (0.22, 0.91)	0.027	0.75 (0.37, 1.48)	0.400	0.77 (0.39, 1.52)	0.400
	P _interaction_	0.652		0.246		0.292	
T2DM	Afflicted	0.22 (0.09, 0.53)	< 0.001	0.37 (0.16, 0.90)	0.028	0.49 (0.22, 1.10)	0.085
	Non-afflicted	0.43 (0.22, 0.81)	0.010	0.69 (0.37, 1.31)	0.300	0.77 (0.40, 1.47)	0.400
	P _interaction_	0.247		0.292		0.238	
General Obesity	Afflicted	0.19 (0.08, 0.48)	< 0.001	0.32 (0.15, 0.71)	0.005	0.38 (0.17, 0.82)	0.013
	Non-afflicted	0.50 (0.24, 1.08)	0.077	0.86 (0.41, 1.77)	0.700	0.90 (0.44, 1.86)	0.800
	P _interaction_	0.144		0.067		0.089	
Central Obesity	Afflicted	0.27 (0.13, 0.53)	< 0.001	0.45 (0.23, 0.90)	0.025	0.52 (0.26, 1.03)	0.059
	Non-afflicted	0.62 (0.24, 1.62)	0.300	0.90 (0.43, 1.90)	0.800	0.87 (0.39, 1.93)	0.700
	P _interaction_	0.176		0.130		0.180	
CKD	Afflicted	0.24 (0.10, 0.54)	< 0.001	0.34 (0.16, 0.76)	0.008	0.42 (0.20, 0.90)	0.026
	Non-afflicted	0,76 (0.35, 1.64)	0.500	1.11 (0.54, 2.28)	0.800	1.13 (0.55, 2.31)	0.700
	P _interaction_	0.073		0.076		0.132	
NAFLD	Afflicted	0.43 (0.20, 0.92)	0.029	0.67 (0.32, 1.40)	0.300	0.76 (0.37, 1.57)	0.500
	Non-afflicted	0.24 (0.09, 0.62)	0.003	0.42 (0.17, 1.04)	0.060	0.43 (0.18, 1.04)	0.062
	P _interaction_	0.391		0.605		0.460	
CVD complicated with T2DM	Afflicted	0.21 (0.05, 0.91)	0.037	0.25 (0.06, 0.95)	0.042	0.23 (0.06, 0.93)	0.039
	Non-afflicted	0.39 (0.21, 0.73)	0.003	0.66 (0.36, 1.19)	0.200	0.73 (0.40, 1.32)	0.300
	P _interaction_	0.468		0.214		0.164	
CVD complicated with Obesity	Afflicted	0.19 (0.04, 0.78)	0.022	0.19 (0.05, 0.66)	0.009	0.20 (0.06, 0.70)	0.012
	Non-afflicted	0.45 (0.23, 0.88)	0.020	0.75 (0.41, 1.39)	0.400	0.79 (0.43, 1.45)	0.500
	P _interaction_	0.316		0.091		0.111	
CVD complicated with CKD	Afflicted	0.30 (0.06, 1.49)	0.14	0.24 (0.05, 1.10)	0.065	0.25 (0.05, 0.97)	0.049
	Non-afflicted	0.43 (0.22, 0.83)	0.012	0.71(0.38, 1.34)	0.300	0.75 (0.40, 1.40)	0.400
	P _interaction_	0.729		0.252		0.332	
CVD complicated with NAFLD	Afflicted	0.25 (0.05, 1.28)	0.10	0.26 (0.06, 1.14)	0.074	0.30 (0.07, 1.34)	0.120
	Non-afflicted	0.39 (0.21, 0.72)	0.003	0.66 (0.37, 1.18)	0.200	0.70 (0.39, 1.24)	0.200
	P _interaction_	0.669		0.370		0.390	

Model 1: Univariate model

Model 2: Adjusted for age, sex, race/ethnicity, marital status, education level, and poverty income ratio (PIR)

Model 3: Additional adjustments included hypertension history, cancer history, alcohol consumption, smoking status, recreational VMPA, work VMPA, commuting VMPA, and sedentary behavior.

**Fig. 3:**
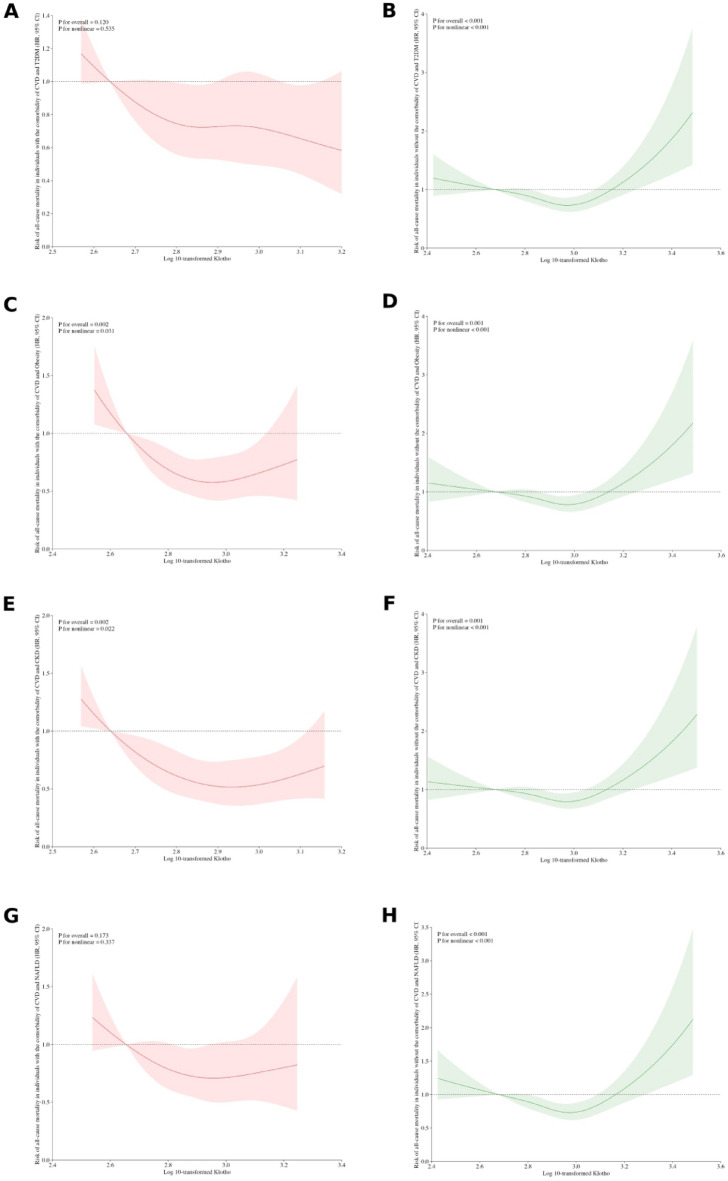
Restricted cubic spline curve for the dose-response relationship of α-Klotho with hazard ratio (HR) of all-cause mortality among participants with (red color) or without (green color) cardiometabolic comorbidities^#^: **(A-B)** CVD complicated with T2DM; **(C-D)** CVD complicated with Obesity;**(E-F)** CVD complicated with CKD;**(G-H)** CVD complicated with NAFLD. ^#^Adjusted for all factors in the full adjustment model. The k1 point was designated as reference point (y = 0).

Moreover, we employed restricted cubic spline (RCS) analyses to explore the nonlinear relationship between Klotho and the hazard ratio (HR) for all-cause mortality in the presence or absence of diverse cardiometabolic comorbidities. In the RCS plots (Fig. 3), the red lines represent the results for afflicted individuals, while the green lines depict those without corresponding comorbidities. Among the population with cardiometabolic comorbidities, Klotho showed an approximately L-shaped dose-response relationship, with an overall trend towards a predominantly negative association with the risk of all-cause mortality. On the contrary, in participants without such comorbidities, the association exhibited a U-shaped pattern, and no significant relationship was found between Klotho levels and all-cause mortality risk. To summarize, in the presence of cardiometabolic comorbidities, Klotho demonstrated a sufficient reduction effect in the risk of all-cause mortality. It underscores the impact of the interplay between Klotho and cardiometabolic diseases on the long-term survival outcomes of individuals.

## Discussion

The study confirmed significant associations between α-Klotho and a reduced risk of cardiometabolic diseases in middle-aged and elderly populations and unveiled non-linear dose-response relationships with T2DM and CKD risks, characterized by U-shaped and L-shaped patterns, respectively. Notably, higher soluble α-Klotho levels were associated with a diminished overall burden of cardiometabolic comorbidities. This relationship between α-Klotho and cardiometabolic comorbidities was particularly evident among individuals with specific sociodemographic profiles: widowed/divorced/separated marital status, non-Hispanic Black ethnicity, low family income, limited educational attainment, history of hypertension, current smoking status, and lower levels of leisure and commuting physical activity but higher work-related physical activity. Moreover, simple mediation analysis revealed that obesity, CKD, and NAFLD acted as intermediaries in the relationship between α-Klotho and CVD risk, with CKD demonstrating the strongest mediating effect. Lastly, regarding long-term effects, the association between α-Klotho and reduced all-cause mortality risk appeared prominent primarily among those with cardiometabolic diseases, rather than non-afflicted population.

While previous studies have examined the relationship between serum α-Klotho and individual diseases, such as cardiovascular disease^33–35^, diabetes^36,37^, chronic kidney disease^38^, non-alcoholic fatty liver disease^39^, obesity^40^, and metabolic syndrome^41^, this study extends this field by investigating the cumulative burden of metabolic comorbidities in the context of cardiovascular disease. By integrating multiple metabolic disorders and CVDs into a unified analytical framework, this study aimed to provide a broader perspective on the potential role of α-Klotho in cardiometabolic health. Our results suggest an inverse association between circulating α-Klotho levels and the cumulative presence of cardiometabolic disorder (CMD) components. Furthermore, through mediation analysis, we explored possible interrelationships among individual CMD components, which may reflect indirect pathways by which α-Klotho could influence cardiovascular outcomes via metabolic processes. Beyond disease prevalence, this study further broadens the scope of α-Klotho research by evaluating its predictive value for all-cause mortality in the CMD-afflicted and non-afflicted populations, highlighting its potential as a biomarker for disease prevention and risk stratification.

The findings indicate Klotho’s protective role in cardiometabolic diseases, aligning with prior research. Preclinical studies on Klotho-knockout mice revealed extensive vascular calcification, endothelial dysfunction, and progressive atherosclerosis^42^. Clinically, elevated serum Klotho levels in adults from the Tuscan community independently lowered the risk of CVD^43^. A study in patients with T2DM also found a negative correlation between Klotho levels and the incidence of coronary heart disease and cerebrovascular accidents^44^. However, there is a limited number of studies systematically and comprehensively evaluating the impact of Klotho on various types of CMDs, especially their concurrent risks, based on large-scale data. This aspect is also a novel contribution to our study. Indeed, mechanistically, Klotho’s protective effects in cardiometabolic diseases are theoretically supported, as CMDs are closely linked to aging processes^45,46^. Furthermore, these cardiometabolic disorders share common underlying factors, often referred to as the “common soil”. These factors include endothelial dysfunction, oxidative stress, inflammation, and abnormalities in glucose and lipid metabolism^47,48^, serving as potential target points for the effects of Klotho. Existing evidence suggests that the protective mechanisms of Klotho against cardiometabolic diseases can be categorized as follows: (1) Klotho protects endothelial cells by boosting endothelial nitric oxide synthase (eNOS) activity, which enhances nitric oxide (NO) production, promoting vasodilation and endothelial cell function^49^. As an enzyme regulating ion channels/transporters, soluble Klotho also can control calcium ion influx stimulated by vascular endothelial growth factor and prevent excessive activity of calcium-dependent proteases, thus preserving endothelial integrity^50^. (2) Alleviating vascular calcification: Klotho may play a functional role in vascular calcification by regulating vitamin C levels^51^, and studies demonstrated that transgenic CKD mice overexpressing Klotho exhibited a slower development of vascular calcification compared to wild-type mice^52^. (3) Anti-endothelial cell apoptosis and senescence: Klotho may interact with the sirtuin (SIRT) epigenetic regulator family, particularly SIRT1, which mediates endothelial cell senescence, inflammation, apoptosis, and other aging-related pathophysiological processes^53^. (4) Inhibiting inflammation and oxidative stress: Klotho deficiency was found to elevate the production of reactive oxygen species (ROS), worsening oxidative stress^54^. Additionally, soluble Klotho can inhibit the activation of NF-κB triggered by pro-inflammatory molecules, such as TNF-α, and decrease the expression of adhesion molecules (ICAM-1 and VCAM-1) on endothelial cell surfaces^55^. (5) Regulating glucose and lipid metabolism: Klotho was identified as a target gene of PPAR-γ, mediating the glucose and lipid metabolism balance^56^, which are crucial in the pathogenesis of CMDs.

Subgroup analysis revealed that the association between Klotho and reduced cardiometabolic comorbidity burden was more pronounced in individuals with higher cardiometabolic risk. In terms of demographic traits, these individuals typically exhibited socioeconomically disadvantaged characteristics, such as being widowed, divorced, or separated, belonging to non-Hispanic Black ethnicity, having low family income, and limited educational attainment. These factors are widely acknowledged as correlating with increased cardiometabolic risk^57–59^. Moreover, prior studies indicate that health disparities linked to socioeconomic status peak during middle and early old age^60^, aligning with the age range of our study population. Notably, the relationship between α-Klotho and cardiometabolic disease (CMD) appears to be particularly pronounced among individuals from lower socioeconomic status (SES) backgrounds, a pattern that warrants further mechanistic exploration. Lower SES is well-documented to be associated with an increased burden of CMD risk factors, including chronic inflammation, oxidative stress, metabolic dysfunction, and vascular abnormalities. These conditions are exacerbated by persistent psychosocial stress, poor nutritional quality, reduced access to healthcare, and heightened environmental exposures—factors that collectively contribute to accelerated disease progression in low SES populations^61–64^. Given the elevated metabolic and cardiovascular risk in low-SES groups, their physiological systems may rely more heavily on Klotho-mediated protective mechanisms, such as modulation of insulin/IGF-1 signaling, anti-inflammatory effects, and oxidative stress regulation^65^. Hence, Klotho may play a compensatory role in mitigating CMD risk, particularly under conditions of heightened metabolic and inflammatory stress. On the other side, Klotho expression is also regulated by systemic stressors, including glucocorticoid dysregulation resulting from chronic hypothalamic-pituitary-adrenal (HPA) axis overactivation^66^, or other hormonal homeostasis disruption^67^, a phenomenon disproportionately observed in low SES individuals experiencing sustained socioeconomic adversity. These pathophysiological disturbances may, in turn, influence endogenous Klotho expression and perturb its downstream signaling pathways^68,69^, potentially altering its cardiometabolic regulatory effects. In other words, low Klotho levels in such environments may lead to a heightened vulnerability to CMD progression, given the combined impact of socioeconomic disadvantages and biological susceptibility. The interaction between SES-related stressors and Klotho insufficiency may amplify CMD risk, leading to faster disease progression and potentially worse clinical outcomes. This aligns with our findings, which suggest that Klotho’s protective effects may be particularly relevant in populations at elevated cardiometabolic risk, rather than in lower-risk groups. Our findings thus underscore the need for targeted preventive strategies that consider both biological and social determinants of health, reinforcing the potential clinical relevance of Klotho as a marker of cardiometabolic resilience in disadvantaged populations. Future research should further explore the precise mechanisms linking socioeconomic adversity, stress physiology, and Klotho-mediated cardiometabolic protection to inform precision medicine approaches tailored to high-risk populations.

Additionally, our findings suggested that Klotho exhibited significant protection against CMDs specifically among current smokers and individuals with hypertension history. Previous studies have highlighted an elevated cardiovascular disease risk among T2DM patients who are current smokers, resulting in a heightened risk of fatal or non-fatal myocardial infarction or sudden death, and cessation of smoking has been shown to reduce mortality risk^70^. Similarly, hypertension was also considered to be closely associated with cardiometabolic risk^71^. Notably, our study also found an intriguing trend where individuals who participated in lower levels of leisure-time physical activity (PA), commuting physical activity, and sedentary behavior, but higher levels of work-related physical activity, tended to derive greater benefits from Klotho’s protective effects against cardiometabolic diseases. As for the relationship between domain-specific physical activity and cardiometabolic risk, the interesting concept of the “physical activity paradox” is necessarily to be introduced^72^. The “physical activity paradox” suggests that leisure-time physical exercise benefits cardiorespiratory fitness and cardiovascular health, whereas work-related physical activity is associated with fatigue, inadequate recovery, elevated 24-hour blood pressure and heart rate due to sustained lifting or static postures, as well as increased stress and inflammation levels, consequently undermining cardiovascular health^73^. On the other hand, considering the characteristics of these domain-specific physical activities, we may deduce that the occupational composition of this group tends to be more inclined towards blue-collar workers engaged in strenuous physical labor. This appears to align with the mentioned socioeconomic status. Overall, these findings suggest that prioritizing the monitoring of Klotho levels in high-risk populations for cardiometabolic diseases or implementing targeted Klotho therapy for such individuals could potentially lead to greater clinical benefits.

Furthermore, the mediation analysis revealed that chronic kidney disease (CKD), as a form of metabolic abnormality, may play a significant mediating role between Klotho and CVD, consistent with prior evidence. On one hand, CKD is associated with various cardiovascular complications such as atherosclerosis, arrhythmias, heart failure, and cardiac fibrosis^74^. On the other hand, since Klotho is predominantly produced in the kidneys, its levels decline with decreasing kidney function^75^. In normal physiological conditions, the kidney is the key regulator of serum Klotho levels^76^. Studies have demonstrated that higher serum levels of Klotho are associated with reduced cardiovascular event risk and mortality during chronic dialysis^77^. Dysregulation of the Klotho axis can heighten cardiovascular risk, increasing the incidence of cardiovascular events in CKD patients^78^. Nevertheless, some research argued that the negative association between α-Klotho and atherosclerosis in diabetic patients was independent of CKD presence^79,80^. Therefore, more prospective studies are warranted to elucidate this complex relationship and its underlying mechanisms in the future.

In terms of long-term effects, compared to non-afflicted individuals, the association between Klotho and reduced all-cause mortality risk was more pronounced in patients with cardiometabolic diseases. We can infer that Klotho possibly extends lifespan and exerts anti-aging effects by improving common pathogenic mechanisms of cardiometabolic abnormalities. These findings align with previous research, including a study indicating that low levels of Klotho are linked to increased risk of cardiovascular death or heart failure hospitalization in stable ischemic heart disease patients^81^. Additionally, single-nucleotide polymorphisms m in the KL gene encoding klotho that was linked to lower plasma klotho levels was associated with increased mortality^82^. Nevertheless, prospective studies addressing the prognosis of cardiometabolic diseases comprehensively are limited, warranting future clinical trials targeting these patients using Klotho as an intervention to gather further evidence supporting this conclusion.

Using large-scale, multi-center, multi-ethnic general population data on middle-aged and older individuals, our study represents the first comprehensive assessment of the relationship between Klotho and CMDs. By considering comorbidities, we provide insights that closely reflect real-world disease complexities. Furthermore, through the integration of Cox analysis for survival cohort data, we enhance our understanding of the long-term implications of this relationship. Notably, our findings underscore the population-specific nature of Klotho’s potential cardiometabolic protective role, laying a foundation for precision medicine practices tailored to high-risk populations. From a clinical perspective, with the emergence of soluble α-Klotho protein administration and strategies to promote endogenous Klotho generation therapy, our study holds substantial potential for clinical translation. Overall, it offers valuable insights for the targeted use of Klotho in the treatment of CMDs, promising advancements in personalized medicine approaches to improve patient outcomes.

Nevertheless, it’s essential to acknowledge several limitations that may affect the interpretation and generalization of our results. Firstly, while our study comprehensively evaluated the relationship between Klotho and CMD prevalence, the observational nature of the study design precludes us from establishing causality. Secondly, despite adjusting for various confounding factors, residual confounding and unmeasured variables may still exist, potentially influencing our results. For example, treatment accessibility, adherence, and broader healthcare disparities across regions and ethnic groups may affect the relationship between Klotho and CMD. Due to the inherent limitations of the available data, future research is needed to further investigate these potential influencing factors. Additionally, the study relied on self-reported data for certain variables, introducing the possibility of recall bias or misclassification. Furthermore, although mediation analysis has offered insights into the intricate interplay among Klotho, CVD, and metabolic disorders, the causal pathways that underlie these relationships remain speculative. Further investigation through interventional studies with rigorous and nuanced designs is needed to elucidate these mediation pathways.

## Conclusion

In conclusion, this study highlights the association between higher α-Klotho levels and a lower burden of cardiometabolic comorbidities, particularly among high-risk cardiovascular population with lower socioeconomic status and unfavorable lifestyles. Moreover, Additionally, our findings suggest potential long-term benefits of Klotho in reducing all-cause mortality among patients afflicted with CMDs, contrasting with non-afflicted individuals. These findings underscore the therapeutic promise of Klotho in managing cardiometabolic diseases and highlight the significance of personalized interventions for high-risk populations. Besides, targeting Klotho could offer a potential strategy to mitigate adverse prognoses among patients with cardiometabolic comorbidities.

### Ethics approval and consent to participate

The study was performed according to the guidelines of the Helsinki Declaration. The original protocol of the NHANES survey adhered to the STROBE statement and was approved by the Ethics Review Committee of the National Center for Health Statistics. NHANES has obtained written informed consent from all participants. Detailed approved code and dates can be found at: https://www.cdc.gov/nchs/nhanes/about/erb.html.

### Statistical analysis

To ensure nationally representative results, we implemented an appropriate weighting methodology to accommodate the intricate NHANES sampling design, as prescribed by the NHANES Guidelines^83^. We utilized NHANES MEC weights for this analysis, adjusting for nonresponse, noncoverage, and unequal probabilities. Serum α-Klotho distributions were right-skewed and log-transformed (base 10) to reduce skewness^84^. The log-transformed α-Klotho value was included as a continuous variable and categorized into four groups based on quartiles (Q1: ≤ 25th percentile, Q2: > 25 to 50th percentile, Q3: > 50 to 75th percentile, Q4: > 75th percentile). The first quartile served as the reference value and was tested for trends. Following the STROBE statement, this study employed fully adjusted, minimally adjusted, and unadjusted models as follows: Model 1: Univariate model; Model 2: Adjusted for age, sex, race, marriage status, education level, and poverty income ratio (PIR); Model 3 (fully adjusted): Additional adjustments included hypertension history, cancer history, alcohol consumption, smoking status, recreational VMPA, work VMPA, commuting VMPA, and sedentary behavior. Weighted Logistic regression models assess the association between serum α-Klotho levels and the prevalence of individual cardiometabolic diseases separately, calculating odds ratios (ORs) and 95% confidence intervals (CIs) in each model. Ordered logistic regression is employed to evaluate the relationship between Klotho and the combined burden and accumulated risk of CVD complicated with metabolic disorders. Subgroup analyses are conducted based on various demographic characteristics, lifestyle, and other medical conditions. To assess the potential mediating role of metabolic disorders in the association between α-Klotho and CVD, we conducted both simple and multiple mediation analyses.

For simple mediation, we applied the counterfactual framework using ‘mediation’ R package. A two-step approach was employed: (1) a logistic regression model was fitted for the mediator, adjusting for covariates; (2) another logistic model was constructed for CVD, incorporating both the exposure (α-Klotho) and the mediator. The indirect effect, direct effect, and proportion mediated were estimated using 1,000 bootstrap resamples^85^. For multiple mediation, we used structural equation modeling (SEM) via the ‘lavaan’ R package, integrating parallel and serial pathways involving NAFLD, obesity, T2DM, and CKD. The WLSMV estimator, suitable for categorical variables, was applied. To account for NHANES’ complex survey design, post-estimation adjustments were made using ‘lavaan.survey’. Additionally, Cox proportional hazards model was used to investigate the association between serum α-Klotho levels and all-cause mortality among populations with the presence or absence of cardiometabolic abnormalities, calculating hazard ratios (HRs) and 95% confidence intervals (CIs). In addition, weighted restricted cubic splines (RCS), with four nodes, were used to evaluate nonlinear relationships, utilizing the “rms” and “RCSplot” packages in R. All statistical analyses were carried out using R (version 4.2.0, http://www.R-project.org), with statistical significance set at a two-sided *p* < 0.05.

## Electronic supplementary material

Below is the link to the electronic supplementary material.

Supplementary Material 1

## Data Availability

The data sets used and/or analyzed during this study are available from the National Health and Nutrition Examination Survey (NHANES) database https://www.cdc.gov/nchs/nhanes/index.htm.

## References

[1] ReFaey, K. et al. Cancer mortality rates increasing vs cardiovascular disease mortality decreasing in the world: future implications. *Mayo Clin. Proc. Innov. Qual. Outcomes*. **5** (3), 645–653 (2021). 34195556 10.1016/j.mayocpiqo.2021.05.005 PMC8240359

[2] Roth, G. A., Mensah, G. A. & Fuster, V. The global burden of cardiovascular diseases and risks: A compass for global action. *J. Am. Coll. Cardiol.* **76** (25), 2980–2981 (2020). 33309174 10.1016/j.jacc.2020.11.021

[3] DALYs, G. B. D. & Collaborators, H. Global, regional, and National disability-adjusted life-years (DALYs) for 359 diseases and injuries and healthy life expectancy (HALE) for 195 countries and territories, 1990–2017: a systematic analysis for the global burden of disease study 2017. *Lancet* **392** (10159), 1859–1922 (2018). 30415748 10.1016/S0140-6736(18)32335-3 PMC6252083

[4] Cosentino, F. et al. Cardiometabolic risk management: insights from a European society of cardiology cardiovascular round table. *Eur. Heart J.* **44** (39), 4141–4156 (2023). 37448181 10.1093/eurheartj/ehad445

[5] Glynn, L. G. Multimorbidity: another key issue for cardiovascular medicine. *Lancet* **374** (9699), 1421–1422 (2009). 19854371 10.1016/S0140-6736(09)61863-8

[6] Oishi, Y. & Manabe, I. Organ system crosstalk in cardiometabolic disease in the age of Multimorbidity. *Front. Cardiovasc. Med.* **7**, 64 (2020). 32411724 10.3389/fcvm.2020.00064 PMC7198858

[7] Einarson, T. R., Acs, A., Ludwig, C. & Panton, U. H. Prevalence of cardiovascular disease in type 2 diabetes: a systematic literature review of scientific evidence from across the world in 2007–2017. *Cardiovasc. Diabetol.* **17** (1), 83 (2018). 29884191 10.1186/s12933-018-0728-6 PMC5994068

[8] Collaborators, G. B. D. R. F. Global burden of 87 risk factors in 204 countries and territories, 1990–2019: a systematic analysis for the global burden of disease study 2019. *Lancet* **396** (10258), 1223–1249 (2020). 33069327 10.1016/S0140-6736(20)30752-2 PMC7566194

[9] Lechner, K. & Krauss, R. M. Obesity and cardiovascular disease: beyond body weight and energy balance. *Eur. J. Prev. Cardiol.* **29** (17), 2216–2217 (2022). 36136860 10.1093/eurjpc/zwac220

[10] Correa, M. M., Thume, E., De Oliveira, E. R. & Tomasi, E. Performance of the waist-to-height ratio in identifying obesity and predicting non-communicable diseases in the elderly population: A systematic literature review. *Arch. Gerontol. Geriatr.* **65**, 174–182 (2016). 27061665 10.1016/j.archger.2016.03.021

[11] Hemmelgarn, B. R. et al. Overview of the Alberta kidney disease network. *BMC Nephrol.* **10**, 30 (2009). 19840369 10.1186/1471-2369-10-30 PMC2770500

[12] Zeb, I. et al. Nonalcoholic fatty liver disease and incident cardiac events: the Multi-Ethnic study of atherosclerosis. *J. Am. Coll. Cardiol.* **67** (16), 1965–1966 (2016). 27102512 10.1016/j.jacc.2016.01.070

[13] Allen, A. M. et al. Nonalcoholic fatty liver disease incidence and impact on metabolic burden and death: A 20 year-community study. *Hepatology* **67** (5), 1726–1736 (2018). 28941364 10.1002/hep.29546 PMC5866219

[14] Ekstedt, M. et al. Fibrosis stage is the strongest predictor for disease-specific mortality in NAFLD after up to 33 years of follow-up. *Hepatology* **61** (5), 1547–1554 (2015). 25125077 10.1002/hep.27368

[15] Kuro-o, M. et al. Mutation of the mouse Klotho gene leads to a syndrome resembling ageing. *Nature* **390** (6655), 45–51 (1997). 9363890 10.1038/36285

[16] Liu, Y. & Chen, M. Emerging role of alpha-Klotho in energy metabolism and cardiometabolic diseases. *Diabetes Metab. Syndr.* **17** (10), 102854 (2023). 37722166 10.1016/j.dsx.2023.102854

[17] Wang, Y. & Sun, Z. Current Understanding of Klotho. *Ageing Res. Rev.* **8** (1), 43–51 (2009). 19022406 10.1016/j.arr.2008.10.002 PMC2637560

[18] Dalton, G. D., Xie, J., An, S. W. & Huang, C. L. New insights into the mechanism of action of soluble Klotho. *Front. Endocrinol. (Lausanne)*. **8**, 323 (2017). 29250031 10.3389/fendo.2017.00323 PMC5715364

[19] Martin-Nunez, E. et al. Klotho expression in peripheral blood Circulating cells is associated with vascular and systemic inflammation in atherosclerotic vascular disease. *Sci. Rep.* **12** (1), 8422 (2022). 35590090 10.1038/s41598-022-12548-z PMC9120199

[20] Cai, S. et al. Relationship between urinary bisphenol a levels and cardiovascular diseases in the U.S. Adult population, 2003–2014. *Ecotoxicol. Environ. Saf.* **192**, 110300 (2020). 32058166 10.1016/j.ecoenv.2020.110300

[21] Wang, J., Liu, F., Kong, R. & Han, X. Association between Globulin and diabetic nephropathy in Type2 diabetes mellitus patients: A Cross-Sectional study. *Front. Endocrinol. (Lausanne)*. **13**, 890273 (2022). 35898464 10.3389/fendo.2022.890273 PMC9311329

[22] American Diabetes, A. Standards of medical care in diabetes–2013. *Diabetes Care*. **36** (Suppl 1), S11–66 (2013). 23264422 10.2337/dc13-S011 PMC3537269

[23] Kushner, R. F. & Ryan, D. H. Assessment and lifestyle management of patients with obesity: clinical recommendations from systematic reviews. *JAMA* **312** (9), 943–952 (2014). 25182103 10.1001/jama.2014.10432

[24] Yin, B. et al. Non-linear association of atherogenic index of plasma with insulin resistance and type 2 diabetes: a cross-sectional study. *Cardiovasc. Diabetol.* **22** (1), 157 (2023). 37386500 10.1186/s12933-023-01886-5 PMC10311747

[25] Levey, A. S. et al. A new equation to estimate glomerular filtration rate. *Ann. Intern. Med.* **150** (9), 604–612 (2009). 19414839 10.7326/0003-4819-150-9-200905050-00006 PMC2763564

[26] Chen, T. K., Knicely, D. H. & Grams, M. E. Chronic kidney disease diagnosis and management: A review. *JAMA* **322** (13), 1294–1304 (2019). 31573641 10.1001/jama.2019.14745 PMC7015670

[27] Lee, J. H. et al. Hepatic steatosis index: a simple screening tool reflecting nonalcoholic fatty liver disease. *Dig. Liver Dis.* **42** (7), 503–508 (2010). 19766548 10.1016/j.dld.2009.08.002

[28] Missel, A. L. et al. Association between fasting insulin and C-reactive protein among adults without diabetes using a two-part model: NHANES 2005–2010. *Diabetol. Metab. Syndr.* **13** (1), 29 (2021). 33691751 10.1186/s13098-021-00645-4 PMC7944601

[29] Almohamad, M., Krall Kaye, E., Mofleh, D. & Spartano, N. L. The association of sedentary behaviour and physical activity with periodontal disease in NHANES 2011–2012. *J. Clin. Periodontol*. **49** (8), 758–767 (2022). 35634657 10.1111/jcpe.13669

[30] Yang, C. et al. Trends and influence factors in the prevalence, intervention, and control of metabolic syndrome among US adults, 1999–2018. *BMC Geriatr.* **22** (1), 979 (2022). 36536296 10.1186/s12877-022-03672-6 PMC9764589

[31] Christensen, K., Gleason, C. E. & Mares, J. A. Dietary carotenoids and cognitive function among US adults, NHANES 2011–2014. *Nutr. Neurosci.* **23** (7), 554–562 (2020). 30326796 10.1080/1028415X.2018.1533199 PMC6467741

[32] Whelton, P. K. et al. ACC/AHA/AAPA/ABC/ACPM/AGS/APhA/ASH/ASPC/NMA/PCNA Guideline for the Prevention, Detection, Evaluation, and Management of High Blood Pressure in Adults: Executive Summary: A Report of the American College of Cardiology/American Heart Association Task Force on Clinical Practice Guidelines. *Circulation* 2018, 138(17):e426-e483. (2017). 10.1161/CIR.0000000000000597 30354655

[33] Xu, J. P. et al. Associations between serum soluble alpha-Klotho and the prevalence of specific cardiovascular disease. *Front. Cardiovasc. Med.* **9**, 899307 (2022). 35795366 10.3389/fcvm.2022.899307 PMC9251131

[34] Lee, J. et al. Association between serum Klotho levels and cardiovascular disease risk factors in older adults. *BMC Cardiovasc. Disord*. **22** (1), 442 (2022). 36221064 10.1186/s12872-022-02885-2 PMC9552482

[35] Zhu, X. et al. Renal function mediates the association between Klotho and congestive heart failure among Middle-Aged and older individuals. *Front. Cardiovasc. Med.* **9**, 802287 (2022). 35509269 10.3389/fcvm.2022.802287 PMC9058082

[36] Wang, K. et al. Association between serum Klotho levels and the prevalence of diabetes among adults in the united States. *Front. Endocrinol. (Lausanne)*. **13**, 1005553 (2022). 36440221 10.3389/fendo.2022.1005553 PMC9681912

[37] Ciardullo, S. & Perseghin, G. Soluble alpha-Klotho levels, glycemic control and renal function in US adults with type 2 diabetes. *Acta Diabetol.* **59** (6), 803–809 (2022). 35286490 10.1007/s00592-022-01865-4 PMC9085659

[38] Zhang, Z. et al. The association between serum soluble Klotho and chronic kidney disease among Us adults ages 40 to 79 years: Cross-sectional study. *Front. Public. Health*. **10**, 995314 (2022). 36276390 10.3389/fpubh.2022.995314 PMC9582855

[39] Chi, Z. et al. Association between Klotho and non-alcoholic fatty liver disease and liver fibrosis based on the NHANES 2007–2016. *Ann. Hepatol.* **28** (5), 101125 (2023). 37286168 10.1016/j.aohep.2023.101125

[40] Orces, C. H. The Association of Obesity and the Antiaging Humoral Factor Klotho in Middle-Aged and Older Adults. *ScientificWorldJournal* 2022:7274858. (2022). 10.1155/2022/7274858 PMC9433301 36061981

[41] Orces, C. H. The association between metabolic syndrome and the anti-aging humoral factor Klotho in middle-aged and older adults. *Diabetes Metab. Syndr.* **16** (6), 102522 (2022). 35660935 10.1016/j.dsx.2022.102522

[42] Lau, W. L. et al. Vitamin D receptor agonists increase Klotho and osteopontin while decreasing aortic calcification in mice with chronic kidney disease fed a high phosphate diet. *Kidney Int.* **82** (12), 1261–1270 (2012). 22932118 10.1038/ki.2012.322 PMC3511664

[43] Semba, R. D. et al. Plasma Klotho and cardiovascular disease in adults. *J. Am. Geriatr. Soc.* **59** (9), 1596–1601 (2011). 21883107 10.1111/j.1532-5415.2011.03558.x PMC3486641

[44] Pan, H. C., Chou, K. M., Lee, C. C., Yang, N. I. & Sun, C. Y. Circulating Klotho levels can predict long-term macrovascular outcomes in type 2 diabetic patients. *Atherosclerosis* **276**, 83–90 (2018). 30048945 10.1016/j.atherosclerosis.2018.07.006

[45] Emami, M. et al. Accelerated biological aging secondary to cardiometabolic risk factors is a predictor of cardiovascular mortality: A systematic review and Meta-analysis. *Can. J. Cardiol.* **38** (3), 365–375 (2022). 34822967 10.1016/j.cjca.2021.10.012

[46] Corina, A. et al. Effects of aging and diet on cardioprotection and cardiometabolic risk markers. *Curr. Pharm. Des.* **25** (35), 3704–3714 (2019). 31692432 10.2174/1381612825666191105111232

[47] Martin-Timon, I., Sevillano-Collantes, C., Segura-Galindo, A. & Del Canizo-Gomez, F. J. Type 2 diabetes and cardiovascular disease: have all risk factors the same strength? *World J. Diabetes*. **5** (4), 444–470 (2014). 25126392 10.4239/wjd.v5.i4.444 PMC4127581

[48] Koliaki, C., Liatis, S. & Kokkinos, A. Obesity and cardiovascular disease: revisiting an old relationship. *Metabolism* **92**, 98–107 (2019). 30399375 10.1016/j.metabol.2018.10.011

[49] Richter, B., Haller, J., Haffner, D. & Leifheit-Nestler, M. Klotho modulates FGF23-mediated NO synthesis and oxidative stress in human coronary artery endothelial cells. *Pflugers Arch.* **468** (9), 1621–1635 (2016). 27448998 10.1007/s00424-016-1858-x

[50] Kusaba, T. et al. Klotho is associated with VEGF receptor-2 and the transient receptor potential canonical-1 Ca2 + channel to maintain endothelial integrity. *Proc. Natl. Acad. Sci. U S A*. **107** (45), 19308–19313 (2010). 20966350 10.1073/pnas.1008544107 PMC2984167

[51] Hum, J. M. et al. Chronic hyperphosphatemia and vascular calcification are reduced by stable delivery of soluble Klotho. *J. Am. Soc. Nephrol.* **28** (4), 1162–1174 (2017). 27837149 10.1681/ASN.2015111266 PMC5373441

[52] Hu, M. C. et al. Klotho deficiency causes vascular calcification in chronic kidney disease. *J. Am. Soc. Nephrol.* **22** (1), 124–136 (2011). 21115613 10.1681/ASN.2009121311 PMC3014041

[53] Kida, Y. & Goligorsky, M. S. Sirtuins, cell senescence, and vascular aging. *Can. J. Cardiol.* **32** (5), 634–641 (2016). 26948035 10.1016/j.cjca.2015.11.022 PMC4848124

[54] Izbeki, F. et al. Loss of Kitlow progenitors, reduced stem cell factor and high oxidative stress underlie gastric dysfunction in progeric mice. *J. Physiol.* **588** (Pt 16), 3101–3117 (2010). 20581042 10.1113/jphysiol.2010.191023 PMC2956948

[55] Maekawa, Y. et al. Klotho suppresses TNF-alpha-induced expression of adhesion molecules in the endothelium and attenuates NF-kappaB activation. *Endocrine* **35** (3), 341–346 (2009). 19367378 10.1007/s12020-009-9181-3

[56] Zhang, H. et al. Klotho is a target gene of PPAR-gamma. *Kidney Int.* **74** (6), 732–739 (2008). 18547997 10.1038/ki.2008.244

[57] Poole, L., Lazzarino, A. I., Smith, K. J. & Hackett, R. A. The combined effect of socioeconomic position and C-reactive protein for predicting incident cardiometabolic disease: findings from a 14-year follow-up study of the english longitudinal study of ageing (ELSA). *SSM Popul. Health*. **24**, 101520 (2023). 37808231 10.1016/j.ssmph.2023.101520 PMC10550841

[58] Kim, A., Lee, J. A. & Park, H. S. Health behaviors and illness according to marital status in middle-aged Koreans. *J. Public. Health (Oxf)*. **40** (2), e99–e106 (2018). 30020525 10.1093/pubmed/fdx071

[59] Merkin, S. S. et al. Race/ethnicity, neighborhood socioeconomic status and cardio-metabolic risk. *SSM Popul. Health*. **11**, 100634 (2020). 32775593 10.1016/j.ssmph.2020.100634 PMC7397689

[60] House, J. S., Lantz, P. M. & Herd, P. Continuity and change in the social stratification of aging and health over the life course: evidence from a nationally representative longitudinal study from 1986 to 2001/2002 (Americans’ Changing Lives Study). *J Gerontol B Psychol Sci Soc Sci* 60 Spec No 2:15–26. (2005). 10.1093/geronb/60.special_issue_2.s15 16251586

[61] Lam, P. H., Chen, E., Chiang, J. J. & Miller, G. E. Socioeconomic disadvantage, chronic stress, and Proinflammatory phenotype: an integrative data analysis across the lifecourse. *PNAS Nexus*. **1** (4), pgac219 (2022). 36329724 10.1093/pnasnexus/pgac219 PMC9615129

[62] Kraft, P. & Kraft, B. Explaining socioeconomic disparities in health behaviours: A review of biopsychological pathways involving stress and inflammation. *Neurosci. Biobehav Rev.* **127**, 689–708 (2021). 34048858 10.1016/j.neubiorev.2021.05.019

[63] Evans, G. W. & Kantrowitz, E. Socioeconomic status and health: the potential role of environmental risk exposure. *Annu. Rev. Public. Health*. **23**, 303–331 (2002). 11910065 10.1146/annurev.publhealth.23.112001.112349

[64] Etindele Sosso, F. A., Holmes, S. D. & Weinstein, A. A. Influence of socioeconomic status on objective sleep measurement: A systematic review and meta-analysis of actigraphy studies. *Sleep. Health*. **7** (4), 417–428 (2021). 34266774 10.1016/j.sleh.2021.05.005

[65] Olejnik, A., Franczak, A., Krzywonos-Zawadzka, A., Kaluzna-Oleksy, M. & Bil-Lula, I. The Biological Role of Klotho Protein in the Development of Cardiovascular Diseases. *Biomed Res Int* 2018:5171945. (2018). 10.1155/2018/5171945 PMC6323445 30671457

[66] Agorastos, A. & Chrousos, G. P. The neuroendocrinology of stress: the stress-related continuum of chronic disease development. *Mol. Psychiatry*. **27** (1), 502–513 (2022). 34290370 10.1038/s41380-021-01224-9

[67] Guarnotta, V., Amodei, R., Frasca, F., Aversa, A. & Giordano, C. Impact of chemical endocrine disruptors and hormone modulators on the endocrine system. *Int. J. Mol. Sci.* **23**(10). (2022). 10.3390/ijms23105710 PMC9145289 35628520

[68] Luthra, N. S., Clow, A. & Corcos, D. M. The Interrelated Multifactorial Actions of Cortisol and Klotho: Potential Implications in the Pathogenesis of Parkinson’s Disease. *Brain Sci.* **12**(12). (2022). 10.3390/brainsci12121695 PMC9775285 36552155

[69] Prud’homme, G. J., Kurt, M. & Wang, Q. Pathobiology of the Klotho antiaging protein and therapeutic considerations. *Front. Aging*. **3**, 931331 (2022). 35903083 10.3389/fragi.2022.931331 PMC9314780

[70] Pan, A., Wang, Y., Talaei, M. & Hu, F. B. Relation of smoking with total mortality and cardiovascular events among patients with diabetes mellitus: A Meta-Analysis and systematic review. *Circulation* **132** (19), 1795–1804 (2015). 26311724 10.1161/CIRCULATIONAHA.115.017926 PMC4643392

[71] Tasic, I. & Lovic, D. Hypertension and cardiometabolic disease. *Front. Biosci. (Schol Ed)*. **10** (1), 166–174 (2018). 28930524 10.2741/s506

[72] Holtermann, A., Hansen, J. V., Burr, H., Sogaard, K. & Sjogaard, G. The health paradox of occupational and leisure-time physical activity. *Br. J. Sports Med.* **46** (4), 291–295 (2012). 21459873 10.1136/bjsm.2010.079582

[73] Holtermann, A., Krause, N., van der Beek, A. J. & Straker, L. The physical activity paradox: six reasons why occupational physical activity (OPA) does not confer the cardiovascular health benefits that leisure time physical activity does. *Br. J. Sports Med.* **52** (3), 149–150 (2018). 28798040 10.1136/bjsports-2017-097965

[74] Jankowski, J., Floege, J., Fliser, D., Bohm, M. & Marx, N. Cardiovascular disease in chronic kidney disease: pathophysiological insights and therapeutic options. *Circulation* **143** (11), 1157–1172 (2021). 33720773 10.1161/CIRCULATIONAHA.120.050686 PMC7969169

[75] Kim, H. R. et al. Circulating alpha-klotho levels in CKD and relationship to progression. *Am. J. Kidney Dis.* **61** (6), 899–909 (2013). 23540260 10.1053/j.ajkd.2013.01.024

[76] Lindberg, K. et al. The kidney is the principal organ mediating Klotho effects. *J. Am. Soc. Nephrol.* **25** (10), 2169–2175 (2014). 24854271 10.1681/ASN.2013111209 PMC4178446

[77] Marcais, C. et al. Circulating Klotho associates with cardiovascular morbidity and mortality during Hemodialysis. *J. Clin. Endocrinol. Metab.* **102** (9), 3154–3161 (2017). 28402487 10.1210/jc.2017-00104

[78] Bi, X., Yang, K., Zhang, B. & Zhao, J. The protective role of Klotho in CKD-Associated cardiovascular disease. *Kidney Dis. (Basel)*. **6** (6), 395–406 (2020). 33313060 10.1159/000509369 PMC7706511

[79] Castelblanco, E. et al. Association of alpha-klotho with subclinical carotid atherosclerosis in subjects with type 1 diabetes mellitus. *Cardiovasc. Diabetol.* **21** (1), 207 (2022). 36221075 10.1186/s12933-022-01640-3 PMC9554979

[80] Keles, N. et al. Is serum Klotho protective against atherosclerosis in patients with type 1 diabetes mellitus? *J. Diabetes Complications*. **30** (1), 126–132 (2016). 26601789 10.1016/j.jdiacomp.2015.09.013

[81] Bergmark, B. A. et al. Klotho, fibroblast growth factor-23, and the renin-angiotensin system - an analysis from the PEACE trial. *Eur. J. Heart Fail.* **21** (4), 462–470 (2019). 30773798 10.1002/ejhf.1424 PMC6458082

[82] Ko, G. J. et al. Wdpa: the association of Klotho gene polymorphism with the mortality of patients on maintenance dialysis. *Clin. Nephrol.* **80** (4), 263–269 (2013). 23993164 10.5414/CN107800

[83] Division of the National Health and Nutrition Examination Surveys. The National Health and Nutrition Examination Survey (NHANES) Analytic and Reporting Guidelines;. (2018).

[84] Kim, D. et al. Association of alpha-klotho and lead and cadmium: A cross-sectional study. *Sci. Total Environ.* **843**, 156938 (2022). 35753483 10.1016/j.scitotenv.2022.156938

[85] Feng, Q. et al. The role of body mass index in the association between dietary sodium intake and blood pressure: A mediation analysis with NHANES. *Nutr. Metab. Cardiovasc. Dis.* **31** (12), 3335–3344 (2021). 34629246 10.1016/j.numecd.2021.08.051

