# Supplementary material for: Anti-aging protein α-Klotho is potential for reducing comorbidity risk of cardiometabolic diseases in vulnerable populations and enhancing long-term prognosis
Source: Sci Rep. 2025 May 14;15:16722. doi: 10.1038/s41598-025-01580-4 (PMC12078659; doi:10.1038/s41598-025-01580-4)
Supplement: Supplementary file 1 — Supplementary Material 1 [file 41598_2025_1580_MOESM1_ESM.pdf]

Supplementary materials

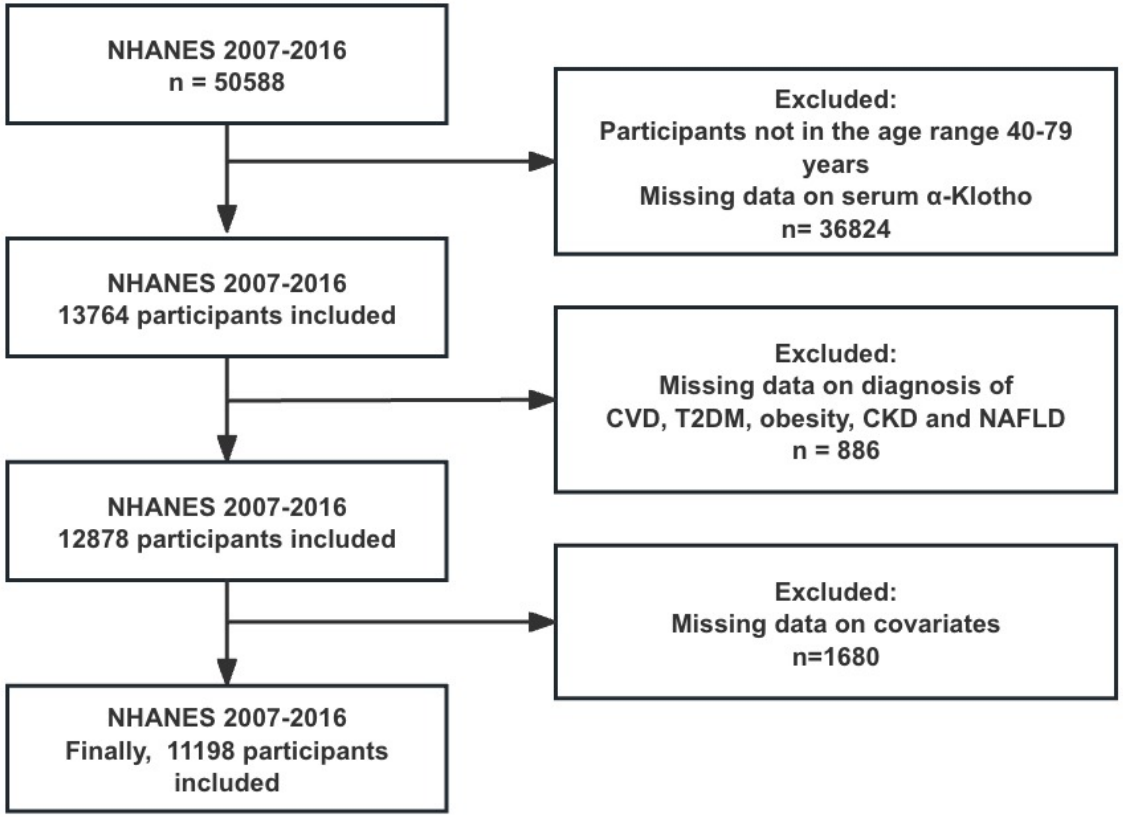

Supplementary Figure 1. Flow chart of the participant selection

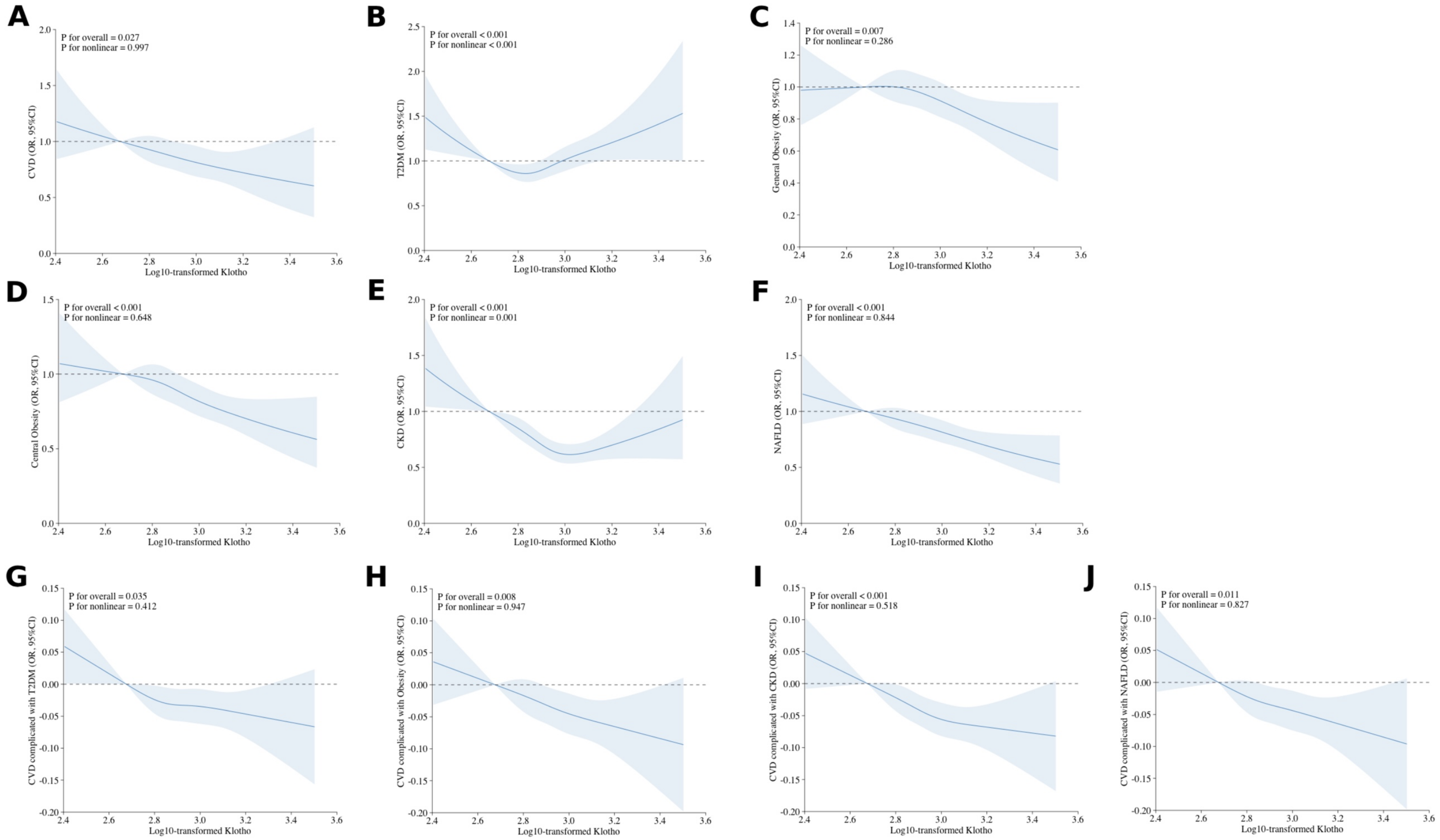

Supplementary Figure 2. Restricted cubic spline curve for the dose–response relationship of α-Klotho with odds ratio (OR) of cardiometabolic disease prevalence# **(A)** CVD; **(B)** T2DM;**(C)** General Obesity;**(D)** Central Obesity;**(E)** CKD;**(F)** NAFLD;**(G)** CVD complicated with T2DM;**(H)** CVD complicated with Obesity;**(I)** CVD complicated with CKD; **(J)** CVD complicated with NAFLD

#Adjusted for all factors in full adjustment model. The k1 point was designated as reference point (y=0)

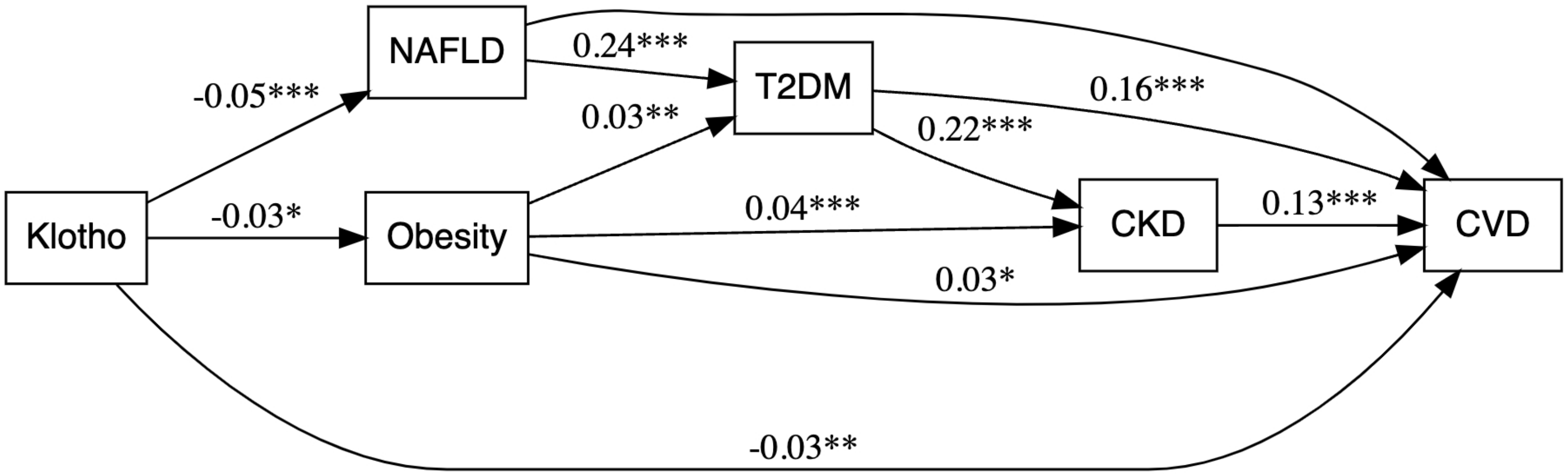

Supplementary Figure 3. A multiple mediating model illustrating the association between α-Klotho and CVD through T2DM, obesity, CKD, and NAFLD.

*All path coefficients are standardized. Only statistically significant paths are labeled with their corresponding coefficients and P-values. Obesity refers to either central obesity or general obesity. Klotho refers to log10-transfromed α-Klotho level. Abbreviations: CVD, cardiovascular disease; T2DM, type 2 diabetes mellitus; CKD, chronic kidney disease; NAFLD, non-alcoholic fatty liver disease; DE, direct effect; IE, indirect effect; TE, total effect. Significance levels: * P < 0.05; **P < 0.01; *** P<0.001.*

Supplementary Table 1. The association between α-Klotho (log10-transformed) and HbA1c, CRP, LDL and HDL using linear regression

Outcomes	Model 1*		Model 2*		Model 3*	
	β (95%CI)	P value	β (95%CI)	P value	β (95%CI)	P value
**HbA1c**	0.35 (0.16, 0.53)	<0.001	0.42 (0.24, 0.60)	<0.001	0.34 (0.16, 0.52)	<0.001
**CRP**	-0.27 (-0.42, -0.12)	<0.001	-0.32 (-0.47, -0.16)	<0.001	-0.33 (-0.52, -0.14)	0.004
**LDL cholesterol**	-3.98(-14.06, 6.10)	0.434	-5.67 (-15.79, 4.46)	0.268	-4.10 (-14.32, 6.15)	0.427
**HDL cholesterol**	1.81 (-2.09, 5.71)	0.358	-0.41 (-3.88, 3.05)	0.813	3.07(-0.05, 6.20)	0.054

**Model 1: Univariate model*

**Model 2: Adjusted for age, sex, race/ethnicity, marital status, education level, and poverty income ratio (PIR)*

**Model 3: Additional adjustments included hypertension history, cancer history, alcohol consumption, smoking status, recreational VMPA, work VMPA, commuting VMPA, and sedentary behavior*

Abbreviations: HbA1c, glycated hemoglobin; CRP, C-reactive protein; LDL, low-density lipoprotein cholesterol; HDL, high-density lipoprotein cholesterol; VMPA, vigorous and moderate physical activity.
